# Motion Tracking of Daily Living and Physical Activities in Health Care: Systematic Review From Designers’ Perspective

**DOI:** 10.2196/46282

**Published:** 2024-05-06

**Authors:** Lai Wei, Stephen Jia Wang

**Affiliations:** 1 School of Design The Hong Kong Polytechnic University Hung Hom China (Hong Kong)

**Keywords:** motion tracking, daily living, physical activity, health care application, design, public health, systematic review, mobile phone

## Abstract

**Background:**

Motion tracking technologies serve as crucial links between physical activities and health care insights, facilitating data acquisition essential for analyzing and intervening in physical activity. Yet, systematic methodologies for evaluating motion tracking data, especially concerning user activity recognition in health care applications, remain underreported.

**Objective:**

This study aims to systematically review motion tracking in daily living and physical activities, emphasizing the critical interaction among devices, users, and environments from a design perspective, and to analyze the process involved in health care application research. It intends to delineate the design and application intricacies in health care contexts, focusing on enhancing motion tracking data’s accuracy and applicability for health monitoring and intervention strategies.

**Methods:**

Using a systematic review, this research scrutinized motion tracking data and their application in health care and wellness, examining studies from Scopus, Web of Science, EBSCO, and PubMed databases. The review used actor network theory and data-enabled design to understand the complex interplay between humans, devices, and environments within these applications.

**Results:**

Out of 1501 initially identified studies, 54 (3.66%) were included for in-depth analysis. These articles predominantly used accelerometer and gyroscope sensors (n=43, 80%) to monitor and analyze motion, demonstrating a strong preference for these technologies in capturing both dynamic and static activities. While incorporating portable devices (n=11, 20%) and multisensor configurations (n=16, 30%), the application of sensors across the body (n=15, 28%) and within physical spaces (n=17, 31%) highlights the diverse applications of motion tracking technologies in health care research. This diversity reflects the application’s alignment with activity types ranging from daily movements to specialized scenarios. The results also reveal a diverse participant pool, including the general public, athletes, and specialized groups, with a focus on healthy individuals (n=31, 57%) and athletes (n=14, 26%). Despite this extensive application range, the focus primarily on laboratory-based studies (n=39, 72%) aimed at professional uses, such as precise activity identification and joint functionality assessment, emphasizes a significant challenge in translating findings from controlled environments to the dynamic conditions of everyday physical activities.

**Conclusions:**

This study’s comprehensive investigation of motion tracking technology in health care research reveals a significant gap between the methods used for data collection and their practical application in real-world scenarios. It proposes an innovative approach that includes designers in the research process, emphasizing the importance of incorporating data-enabled design framework. This ensures that motion data collection is aligned with the dynamic and varied nature of daily living and physical activities. Such integration is crucial for developing health applications that are accessible, intuitive, and tailored to meet diverse user needs. By leveraging a multidisciplinary approach that combines design, engineering, and health sciences, the research opens new pathways for enhancing the usability and effectiveness of health technologies.

## Introduction

### Daily Living and Physical Activity in Health Care

Motion tracking data are pivotal for understanding physical activity in hospitalization and rehabilitation [1,2]. However, the procedures for investigating motion tracking data have not yet been systematically reported, especially in the context of user activity recognition in health care application studies. Several findings indicate that living patterns, such as daily activity trajectories, physical activity, and dietary behaviors, are associated with the initial stages of disease development [3-5]. Inadequate daily physical activity exacerbates symptoms of mental distress and contributes to medical illnesses, including cardiovascular and kidney diseases [6,7]. Conversely, regular physical activity can enhance wellness by activating the immune system and reducing inflammation [8,9]. Monitoring activities of daily living (ADLs) [10] is vital for self-care and long-term health [11,12], making the analysis of daily living and physical activity data crucial for disease prevention and promoting a healthy lifestyle.

A comprehensive analysis of motion tracking data in daily living and physical activities scenarios is essential for delving into human activity patterns and behavior intentions. Research shows that activity patterns are linked to behavior intentions [13,14], and motion data help uncover these patterns, improving diagnostic efficiency [15]. The motion data of ADLs represent activity patterns [16,17], while the formation of these patterns is multifaceted [18], with external influences playing a role [19]. Accumulated movement behaviors, when stimulated by the environment, become habitual actions [20,21]. By analyzing the motion data of ADLs, researchers have found that the formation of activity patterns is attributed to individuals’ cognitive representations [22] and environmental factors, such as sedentary behaviors in working and learning spaces [10,23], as well as daily routines at home [24]. Thus, motion tracking data, enriched by environmental context, effectively represent and interpret human behaviors across different settings.

When applied as representations of ADLs, motion data can facilitate behavioral change through interventions. The analysis and visualization of motion data concerning ADLs can potentially enhance mutual understanding between physicians and patients in medical education and remote clinical consultations [25,26]. Patients’ trajectories of past daily activity and medical histories are essential indicators for physicians to understand symptoms [27]. Human physical activity patterns can reflect the formation of disease and aid in the prevention and management of illness conditions [28,29]. In addition, many researchers have devised human activity interventions and suggested that early care physical activity interventions are feasible for promoting healthy lifestyles [30,31].

### Motion Tracking Technology

The evolution of motion tracking technologies in health care has transitioned from basic motion detection systems aimed at rehabilitation to artificial intelligence (AI)–enhanced wearable sensors for personalized care. Early systems focused on simple motion detection, primarily for rehabilitation [1]. Advancements led to the inclusion of wearable sensors and AI for personalized health care [32]. Recent developments have embraced Internet of Things (IoT) frameworks for more comprehensive health monitoring [33]. In addition, the shift toward patient-centered care has been facilitated by technologies that assess and support daily activities and physical functions [11,34]. The integration of machine learning and sensor data fusion has further enhanced the ability to monitor and analyze patient movement in real time, contributing to preventive health care and improved treatment outcomes [12,35]. Researchers in health care and computer science have discussed the possibility of using motion tracking. Data classification, precision, validity, and diagnostic prediction in activity recognition are popular areas of investigation [36,37]. Several studies have also discussed how collecting voluminous data might contribute to personalized health surveillance systems [32,33]. Furthermore, scholars in computing have claimed that high-quality data would enhance data-driven AI processing [38,39]. IoT, with motion tracking techniques (eg, inertial measurement unit, smartphone, smartwatch, Mocap system, etc) [40,41] and AI processing (eg, human activity recognition, positioning system, etc) [35,42], have been widely used. It is anticipated that AI processing using high-quality motion tracking data would provide accurate and timely health information. For instance, smartphones with built-in motion-tracking sensors can record activity trajectories and routine data to identify activity types and patterns [43]. Studies have also found that motion data can be used to learn about humans’ typical behaviors and identify any outlier activities [44,45]. Moreover, it can boost individuals’ likelihood of engaging in physical activity over the long term, which is associated with habit formation [46,47].

However, the data interpretation of human activity trajectories, environment, and process has not been discussed as extensively as technology. Conversely, several studies have reported that learning from health information could promote health literacy [48,49]. Yet, the public has a long-standing misunderstanding of technology [50,51]. The technology-based health information needs to be in readable language [52,53]. In the meantime, the foundational criteria (ie, ease of use, usefulness, and enjoyment) of the technology acceptance model (TAM) provide insights to evaluate human-technology interaction [54,55]. TAM exemplifies the 3 needs of humans in interpreting technology-based health information.

### The Usability of Motion Tracking Data

Designers play a key role in making data legible, efficiently transmitted, and actively engaging for users [56-58]. To optimize the utility and interpretability of motion tracking for both professionals and users, designers should gather health information needs from the user’s cognitive standpoint, thereby refining the functionality and analytical clarity of motion-tracking systems [59,60]. The adoption of data-enabled design (DED) [61-63] in health care emphasizes continuous data refinement and context-specific design. On the basis of these, health care–related applications, as intelligent solutions [62], should integrate data, users, and environments to optimize the transmission of health information and the intelligent ecosystems. Scholars asserted that design studies could illuminate the nature of nonsocial entities, such as data, to create durable systems [64,65]. This approach has been implemented in designing health interventions [66,67]. It overcomes the barrier between people and technology, offering guidance for enhancing the sustainable hybridization of the social and the technical entities.

Motion tracking serves as a bridge, delivering activity data to health care professionals and users. Understanding data collection and analysis is vital, involving motion data (eg, trajectories and durations) and environmental elements in physical settings. Analysis entails identifying and classifying activities. Yet, interactions among researchers, participants, devices, and environments are seldom explored. Therefore, it is critical to methodically study these interactions within health care applications and their environments.

### Integrating Actor Network Theory in Research

In health care application research on ADLs, interactions occur between participants, sensors, cameras, and scenario objects. The actor network theory (ANT) has been instrumental in analyzing these interactions within a network [68,69], especially in human-computer interaction and design studies [70]. ANT outlines three critical aspects for network analysis: (1) actor-led activities, where initiators, such as individuals or devices, lead activities and interact with others; (2) purpose-oriented interactions, which serve as the guiding principle for activities; and (3) the dynamic interplay between devices, environmental factors, and individuals, shaping the network. Integrating ANT into motion tracking data analysis illuminates the movement trajectories and environmental influences, clarifying human-device-environment relationships.

### Objectives

Prior research has thoroughly examined motion tracking in health care, mainly focusing on algorithm validation and technology deployment for rehabilitation and hospitalization [42,71]. Meanwhile, several studies have reviewed the benefits of physical activity for medical purposes [10,26]. However, systematic reviews exploring the use of motion tracking techniques in ADLs and their connection with humans for health care and physical wellness research are scarce. Furthermore, studies investigating the process of analyzing ADLs for health care applications are even rarer. This study shifts focus to motion data, participant engagement, and situational contexts as key components in health care technology. It delves into how participants interact with technology in physical environments, technology adoption, and participant behavior. This systematic review aimed to map and collate literature on the motion tracking data of daily living and physical activities for health care and physical wellness application research. It aimed to evaluate the research landscape, identify literature gaps, and endorse a designer-engaged approach to studying ADLs. The overarching research questions are as follows:

1. How was the motion tracking technique used in ADLs for health care–related studies and physical wellness applications?
2. What are the environmental factors, interactions, and processes of ADLs in health care and physical wellness application research?
3. What is the design opportunity in health care and physical wellness application research?

## Methods

### Overview

Our research focuses on investigating the motion tracking data, interaction, and process of ADLs in health care and physical wellness applications from the designers’ perspective. Owing to the exploratory and descriptive nature of our research question, we opted for a systematic review [72,73], with the aim of compiling and comprehensively summarizing the relevant research. To guarantee rigor and coherence with our research objectives, 2 researchers meticulously screened the studies for inclusion. These 2 researchers independently conducted screening work on the same data set and met weekly for discussions. Following 5 detailed discussions about the screening methodology, centered on eligibility criteria and research focus, we conducted 2 pilot searches to refine our search strategy and ensure the accuracy of our results. In addition, we discussed the framing of questions being addressed with reference to participants, interventions, comparisons, and outcomes (PICO) [74], ensuring a comprehensive and structured approach to our review (Table 1). The overarching goal of the systematic review is to identify and map the available evidence investigating the technology use in everyday health care and physical wellness apps and its relationship with humans and environments. We then applied ANT [68,69] to analyze the interaction in activity scenarios and used design narrative through the lens of DED [62] to analyze the process of motion tracking data in health care and physical wellness application research scenarios.

Given the independent nature of technology, user behaviors, and environments in our research, we developed bespoke criteria for bias assessment. This approach enabled 2 independent reviewers to identify potential biases in study design, execution, and reporting accurately. Discrepancies between reviewers were resolved through consensus, ensuring a balanced evaluation. To further validate our review, we engaged relevant scholars to critique the search strategies of both pilot and final searches stages, substantially enhancing the review’s validity and comprehensiveness. Our thorough assessments of potential biases, both at the study and outcome levels, were integrated into our data synthesis, paving the way for a credible interpretation of the evidence (detailed in Multimedia Appendix 1 [75-128]).

**Table 1:** Search term– and strategy-based participants, interventions, comparisons, and outcomes (PICO) framework and research questions.

Component	Details	Search terms	Search strategies
Population	General public, including special populations such as children, pregnant women, older adults, people with disabilities, and athletes	N/A^a^	N/A
Intervention	Use of motion tracking techniques in ADLs^b^ and physical activity scenarios	Motion tracking	TITLE-ABS-KEY (“motion capture” OR “MoCap” OR “motion analysis” OR “motion tracking” OR “body positioning” OR “human activity recognition” OR “IMU” OR “dynamometry”)
	Application of AI^c^ processing for activity classification and analysis; or using devices (including sensors, motion capture technology, IoT^d^ devices, VR^e^, AR^f^, and MR^g^)	IoT, AI, VR, and AR	TITLE-ABS-KEY (“Internet-of-things” OR “IoT” OR “wearable” OR “virtual reality” OR “augmented reality” OR “mixed reality” OR “machine learning” OR “deep learning” OR “decision making” OR “artificial intelligence” OR “AI”)
Comparator	Variation in technology use, sensor placement, and participant interaction across different studies	N/A	N/A
Outcome	Identification and classification of daily living and physical activities within health care and physical wellness applications Understanding of environmental factors, interactions, and processes in health care and physical wellness applications Exploration of design opportunities in health care and physical wellness applications	Health and sport application	TITLE-ABS-KEY ([“sport” OR “kinematics” OR “sport analytics” OR “wellness” OR “health”] AND [“smart health” OR “healthcare” OR “health monitoring” OR “health of things” OR “digital health” OR “mobile health system” OR “behaviour change” OR “decision making”])

^a^N/A: not applicable.

^b^ADL: activity of daily living.

^c^AI: artificial intelligence.

^d^IoT: Internet of Things.

^e^VR: virtual reality.

^f^AR: augmented reality.

^g^MR: mixed reality.

### Selection Criteria

#### Study Types

Original research articles published in scientific, technical, and medical journals in English from January 2013 to December 2022 were considered. Reviews, conference abstracts, magazines, and newspaper articles were excluded. We concentrated exclusively on studies encompassing ADLs, specifically within the realms of health care and physical wellness application research, to ensure the relevance of motion tracking technology to real-world health and wellness contexts. Eligibility for inclusion was determined for studies using motion-tracking sensors, motion capture technology, IoT devices, multiple sensors capable of motion tracking, virtual reality (VR) or augmented (mixed) reality, or AI, highlighting our focus on advanced technologies that offer innovative approaches to monitoring and enhancing health-related activities. Eligible populations include the general public as well as specific demographics including children, pregnant women, older adults, individuals with disabilities, and athletes. Exclusions were applied to studies that (1) focused on technology or materials development (ie, technical validation), as our interest was in direct applications of technology in health and wellness, rather than preliminary stages of technological development; for instance, one research focused on preliminary technology validation without applying findings to enhance health-related activities, missing our application-focused criteria, although it mentioned motion tracking, smart systems, inertial measurement unit, and ADLs [129]; (2) presented a data set without further analysis, as our aim was to understand the implications of data on ADLs, necessitating detailed data interpretation; (3) investigated activities in clinical scenarios such as injury, impairments, hospitalization, rehabilitation, etc, because our focus was on everyday activities rather than those strictly within clinical settings; (4) were applied to nonhuman subjects to maintain the applicability of findings to human health and wellness; or (5) did not include any user study results or did not clearly explain their findings, as comprehensible and applicable user data are crucial for informing practical health care and wellness interventions (Textbox 1).

**Eligibility criteria for considering the studies in the review.**

**Inclusion criteria**

- Study types
  Original research articles published in scientific, technical, and medical journals in English from January 2013 to December 2022
  Studies that encompass activities of daily living (ADLs) or physical activity within the scope of health care and physical wellness application research
  Studies using motion-tracking sensors, motion capture technology, internet of things (IoT) devices, multiple sensors capable of motion tracking, virtual reality (VR) or augmented (mixed) reality (A[M]R), or artificial intelligence (AI)
- Population
  General public, including special populations such as children, pregnant women, older adults, people with disabilities, and athletes
- Materials
  Research investigating the use of motion tracking for ADLs analysis
  Studies using motion-tracking sensors, motion capture technology, or multiple sensors for motion monitoring, analysis, visualization, or providing feedback
  Research integrating a combination of AI processing, IoT devices, or VR (A[M]R) technologies
  Studies integrating motion tracking data and AI technology for the classification of ADLs
- Comparison
  Variation in technology use, sensor placement, and participant interaction across different studies
- Research outcomes
  Measures or indexes describing the activity in health care or physical wellness application research (eg, daily human activities, physical activities, or daily activities of special populations)
  Studies focusing on the use of motion tracking for monitoring, analyzing, visualizing, or providing feedback
  Research related to health care or physical wellness system design

**Exclusion criteria**

- Study types
  Reviews, conference abstracts, magazines, and newspaper articles
  Studies focused on technology or materials development (technical validation)
  Research presenting a data set without further analysis
  Investigations in clinical scenarios such as injury, impairments, hospitalization, rehabilitation, etc
  Studies applied to nonhuman subjects
  Studies that did not include any user study results or did not clearly explain their findings
- Population
  Investigations in clinical scenarios such as injury, impairments, hospitalization, rehabilitation, etc
  Studies applied to nonhuman subjects
- Materials
  Studies not using the described technologies or not focused on the specified applications within health care and physical wellness
- Research outcomes
  Outcomes not related to the activity in health care or physical wellness application research, or those not providing meaningful insights into monitoring, analysis, visualization, or feedback within these contexts

#### Materials

Health care or physical wellness application research investigated the use of (1) motion tracking for the analysis of ADLs; (2) motion-tracking sensors, motion capture technology, or multiple sensors for motion monitoring, analysis, visualization, or providing feedback in ADL scenarios; (3) a combination of AI processing, IoT devices, or VR or augmented (mixed) reality technologies; or (4) integrating motion tracking data and AI technology for the classification of ADLs.

#### Research Outcomes

Any measure or index that described (1) the activity in health care or physical wellness application research (eg, daily human activities, physical activities, or daily activities of special populations); (2) the use of motion tracking for monitoring, analyzing, visualizing, or providing feedback; and (3) health care– or physical wellness–related system design was included.

#### Search Strategy

**Figure 1:**
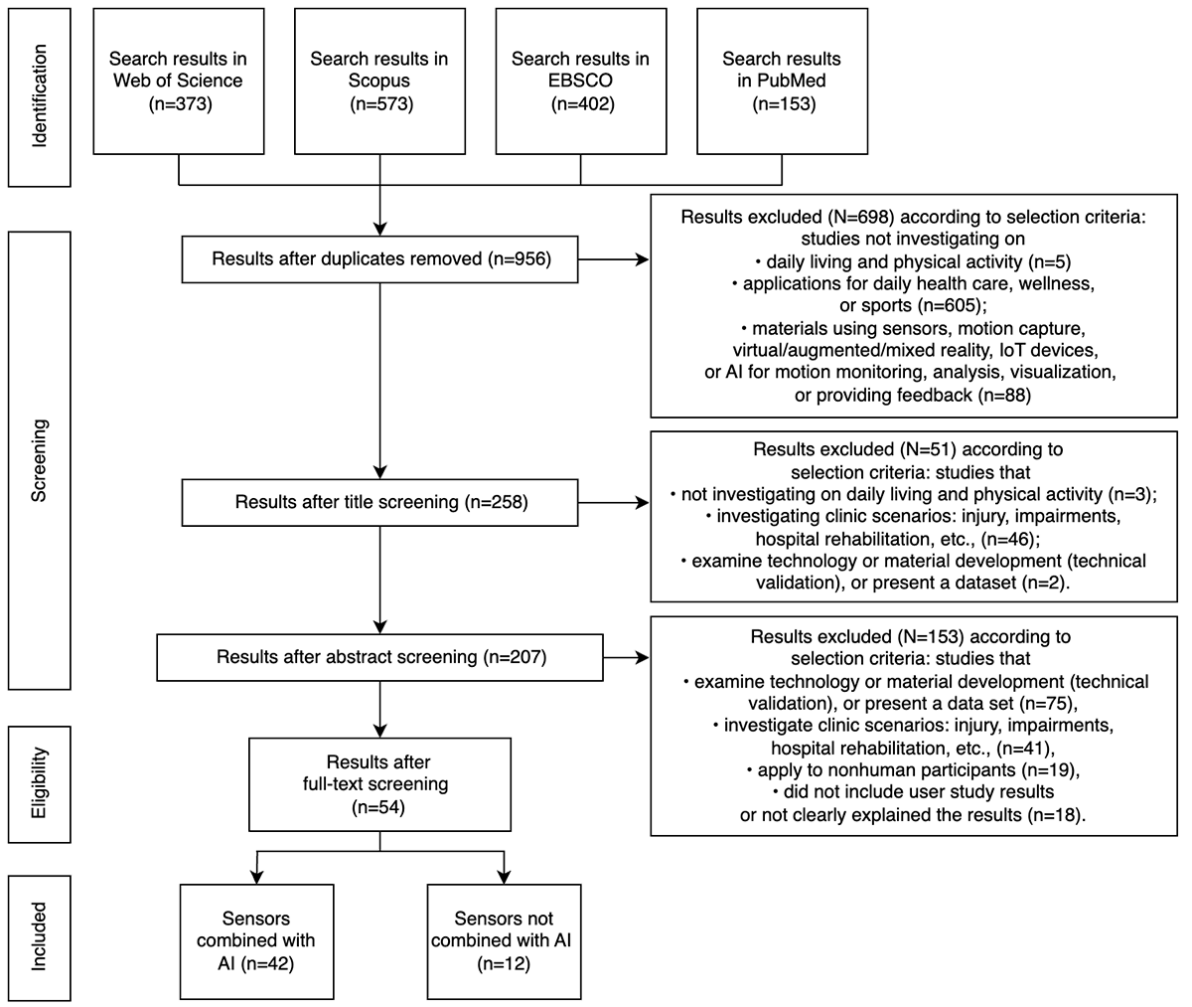
The flowchart of the selection process of articles. AI: artificial intelligence; IoT: Internet of Things.

Two pilot searches were conducted using the Scopus and Web of Science electronic databases in August 2022, applying search strategies detailed in Multimedia Appendix 2. The first search yielded an exceedingly limited number of articles, while the second search displayed a broader range. Upon reviewing the titles and abstracts from the pilot search results, we observed that the terms “physical activity” and “daily living activity” might lead to irrelevant research fields, such as heart rate monitoring and step count. In contrast, “motion tracking” and “motion capture” more accurately capture the essence of physical activity within our research context. These terms are commonly used in the field of motion capture technology research. Consequently, we gave these terms precedence in our investigation, which assisted in the strategic identification of pertinent keywords for inclusion. Moreover, from the results of the initial 2 pilot searches, we identified additional keywords related to health care applications, AI, and motion tracking, such as “health of things” and “dynamometry.” Following discussions, we selected these terms for use in our final search queries (Table 1). The search strategy was subsequently refined and executed in September 2022, covering the following 4 databases: Web of Science, Scopus, EBSCO, and PubMed (Figure 1). The titles, abstracts, and index terms were screened to identify the studies that met our stated eligibility criteria as outlined in Textbox 1.

### Data Extraction

Drawing from previous research in health care and motion tracking, our study builds upon the insights provided by Straczkiewicz et al [42], who illustrated the relationships among different human activity recognition processing phases through data visualizations. Their approach to data visualization reveals the connections among co-occurring factors identified across the reviewed articles over time. In addition, we also considered the work of van Kollenburg et al [62], who explored design narratives for situated design exploration, thereby enriching our understanding of the data analysis process and its application in health care scenarios [62].

**Figure 2:**
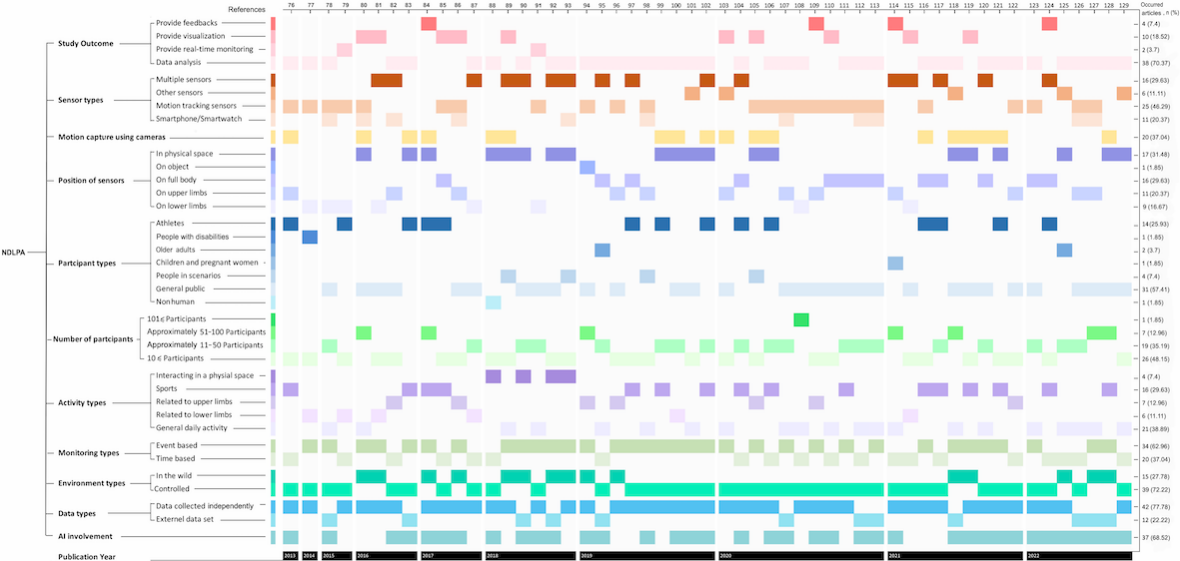
The Network of Daily Living and Physical Activity (NDLPA) among included studies. AI: artificial intelligence. For a higher-resolution version of this figure, see Multimedia Appendix 3.

Through the data extraction process from the selected articles, we organized the data into 11 categories: (1) study outcome (subcategories encompassing providing feedback, visualization, real-time monitoring, and data analysis); (2) sensor types (subcategories include multiple sensors, other sensors, motion-tracking sensors, and smartphone or smartwatch); (3) motion capture using cameras; (4) position of sensors (eg, physical space, on object, full body, upper limbs, or lower limbs); (5) participant types (individuals on whom sensors were placed or who were being monitored, including athletes, people with disabilities, older adults, children, pregnant women, people in scenarios, general public, or nonhuman subjects); (6) number of participants (categorized as >101, 51-100, 10-50, or <10); (7) activity types (covering interactions in physical spaces, sports, activities related to upper or lower limbs, and general daily activities); (8) monitoring types (differentiated by event or time-based monitoring); (9) environment types (distinguished as in the wild or controlled); (10) data types (differentiating between data collected independently or from external data sets); and (11) Al involvement. Then, using the timeline and the categories of the included studies, we provided a publication year–based summary that illustrates the Network of Daily Living and Physical Activity (NDLPA) among included studies, as depicted in Figure 2.

## Results

### Identification of the Studies

**Figure 3:**
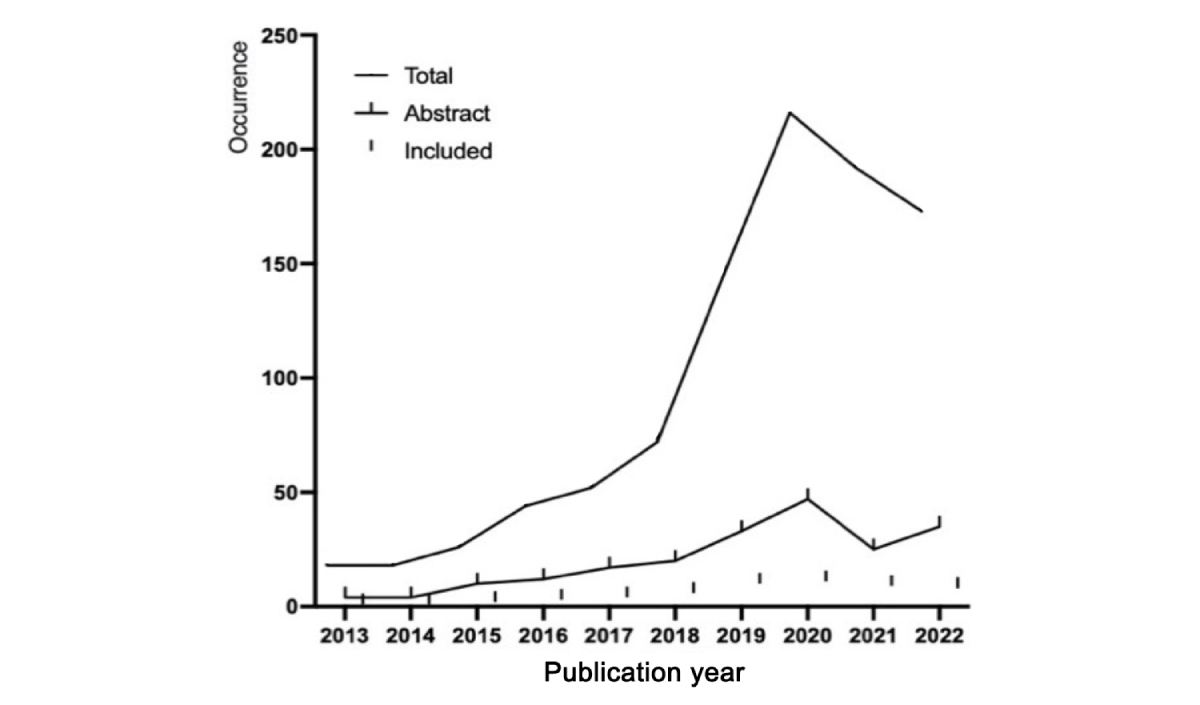
The distribution of total selection, abstract screening, and included articles based on publication year.

The electronic search yielded 1501 studies. After removing internal duplicates, 956 studies remained. On the basis of the eligibility criteria, 258 articles were chosen through title screening (Figure 1). A total of 54 articles were included for full-text appraisal after the abstract screening process [75-128]. The results from database search indicated that the relevant research from 2013 to 2018 grew steadily, while research articles published from 2019 to 2022, aligning with our interests, experienced a significant uptick and consistently maintained a high volume (Figure 3; Table 2). The findings (Figure 2) revealed that 54 articles used motion tracking data in health care and physical wellness applications, of which 42 (78%) articles used motion-tracking sensors [75-81,84-86,88-97,101, 103-116,119,121-123,125,126]. Overall, 70% (38/54) of the articles used AI technology combined with motion tracking data [75,77,79,82-85,87,89-91,93,94,97,99-101,103,105,106,108,109,111-113,117-128]. The role of AI mainly functions for data classification, monitoring, and visualization among the included studies. Furthermore, 80% (43/54) of the studies recruited participants for user study [75,76,78-81,83-88,90,92,93,95-105,107-110, 113-116,118-124,127,128]. Twelve articles used public data set [78,82,89,91,94,106,111,112,117,125-127]. Most of the public data set that were used are University of California, Irvine human activity recognition, center of advanced studies in adaptive system, mobile health, University of Milano Bicocca smartphone-based human activity recognition, mobile sensor data anonymization, a large multipurpose human motion and video data set, and wireless sensor data mining data sets.

**Table 2:** The distribution of total selection, abstract screening, and included articles.

Publication year	Total (n=956), n (%)	Abstract screening (n=207), n (%)	Included articles (n=54), n (%)
2013	18 (1.9)	4 (1.9)	1 (1.8)
2014	18 (1.9)	4 (1.9)	1 (1.8)
2015	26 (2.7)	10 (4.8)	2 (3.6)
2016	44 (4.6)	12 (5.8)	4 (7.3)
2017	52 (5.4)	17 (8.2)	4 (7.3)
2018	72 (7.5)	20 (9.7)	6 (10.9)
2019	145 (15.2)	33 (15.9)	9 (16.4)
2020	216 (22.6)	47 (22.7)	11 (20)
2021	192 (20.1)	25 (12.1)	9 (16.4)
2022	173 (18.1)	35 (16.9)	7 (13)

### Descriptions of Technology Use, Participants, Activity, and Outcomes

#### Overview

The contributing studies displayed a broad spectrum of methodologies, participant types, and technology uses. Characteristics of activity varied from studies focusing on lower limb activities to those analyzing full-body movements, using a range of sensors and AI technologies. Most studies used a combination of accelerometers, gyroscopes, and sometimes cameras across diverse settings from controlled environments to in-the-wild scenarios. Participant types ranged from the general public, including specific groups such as athletes and older adults, to nonhuman participants in a few instances.

#### Technology Use

Most of the included publications (42/54, 78%) used technology based on AI combined with motion tracking techniques or multiple sensors. Among those using sensors, studies incorporating accelerometer and gyroscope sensors formed the core of the investigations (42/54, 78%), including those using a smartphone or smartwatch (11/54, 20%) and those using multiple sensors capable of motion tracking (16/54, 30%). Fewer studies used other types of sensors (eg, temperature sensors, light sensors, etc; 6/54, 11%) or combined them with motion capture using cameras (10/54, 19%). Moreover, 17% (9/54) of the studies exclusively used cameras for motion capture. Details are provided in Figure 2. A combination of accelerometer and gyroscope sensors has been deemed essential for tracking everyday human activity over the past decade. They were used to collect data on typical ADLs, such as standing, walking, sitting, and jumping, while ambient sensors were used less frequently. Physical activities such as jumping [75,78,98], playing basketball [96,116], soccer [83], baseball [115], running [105], ballet dance [103] skiing [127], karate [84], and Taijiquan [123] were studied. Meanwhile, AI processing in the included articles functions for data extraction and classification to achieve daily living and physical activities recognition.

According to the studies using motion tracking data and classifiers, using a minimal pair of accelerometer and gyroscope sensors to acquire motion tracking data can achieve activity recognition with high precision [75,77,78,81,85,97,107,108, 113,121]. Several scholars have suggested that the human activity recognition system has the potential to enhance the efficiency of health care applications using multiple pairs of accelerometer and gyroscope sensors [94,101]. Most studies obtained original motion tracking data of ADLs for evaluating health care systems, whereas fewer studies adopted public data sets for activity recognition applications.

#### Position of Sensors

The method of sensor placement among the included 54 studies was distributed relatively evenly (Figure 2). The sensor placements in most of the included articles were implemented on the full body (n=16, 30%) and in physical spaces (n=17, 31%), with relatively fewer studies focusing on the upper limb (n=11, 20%) and the lower limb (n=9, 17%). However, 1 (2%) article reported that the sensors were placed on an object. The findings suggest that the placement of sensors is inherently determined by the nature of the activities. ADLs were categorized into lower limb–based activities (eg, walking, standing, sitting, etc); upper limb activities (eg, eating, talking on the phone, washing dishes, cooking, etc); and full-body activities (eg, playing basketball, dancing, skiing, etc). Therefore, sensor placements are specifically tailored to match the inherent types of activities being tracked.

The studies that involved placing sensors on the full body and in environments were widely reported [84,94,96,103,109-112, 115,116,119,122,123], and the purposes of using full-body motion tracking are commonly argued for analyzing specific movements or securing the credibility of system evaluations [94,106,110,112,115,116,123]. The investigations involving the placement of sensors in physical spaces mostly use motion capture and image processing techniques [117,119]. Researchers have placed high-speed cameras to capture movements during sports activities [75,82-84,88,98,101,105,115,120,127] and in specialized work situations, such as maritime occupations [104]. One study [93] placed accelerometer and gyroscope sensors on an object, specifically a water container, to determine the frequency and amount of use from the container’s motion tracking data. The positioning of sensors has become increasingly precise and intricate over time. Research has primarily targeted the upper limb, full body, or physical spaces.

#### Participants

The research exploring motion tracking of daily living and physical activities for health care applications has studied a variety of participant types (Figure 2), including the general public (healthy individuals; 31/54, 57%), people in specialized scenarios (4/54, 7%), children and pregnant women (1/54, 2%), older adults (2/54, 4%), people with disabilities (1/54, 2%), and athletes (14/54, 26%) over the past decade. One exception involved using a male cadaver for the analysis of vehicle-cadaver tests. From the results, researchers primarily applied motion capture technology to healthy individuals aged between 18 and 60 years and to athletes. Activities involving healthy individuals can provide representative activity characteristics for training activity recognition models and identifying outliers in activities through activity classification. In the last 3 years, researchers have increasingly prioritized the use of data collected from the public and youths for sports education–related research.

The number of participants recruited varied significantly across the included studies. Approximately half of the selected studies collected data from <10 participants (26/54, 48%), and a smaller number gathered data from 10 to 50 participants (19/54, 35%). Moreover, 13% (7/54) of the studies collected data from 50 to 100 individuals. Half (27/54, 50%) of the studies used 10 to 100 samples from public databases [77,89,94,106,111,112,117,125-127]. Only one study [107] amassed gait data from >1000 healthy individuals. The quantity of the sample size correlates with the types of participants and activities. In sports-related activities, the sample size ranged from 1 to 50 individuals, predominantly involving athletes. For studies on daily living activities, the sample size varied from 10 to 1000s of individuals, primarily focusing on the healthy general public.

#### Activity

Prior studies have classified activities according to the intensity of exercise [130]. In this study, the activity classification extends the previous categorization and is divided into general daily activity (21/54, 39%; eg, walking, standing, sitting, etc); activities related to the lower limb (6/54, 11%; eg, running, jumping, etc); activities related to the upper limb (7/54, 13%; eg, drinking, writing, cutting, etc); sports (16/54, 30%; eg, playing basketball or baseball, dancing, Taijiquan, etc); and interaction in a physical space (4/54, 7%; eg, living in a laboratory setting house, farming, etc) based on the sensor placements and the purposes of the activities (Figure 2). Most studies (39/54, 72%) specified that they were conducted in laboratory experiment settings and aimed to minimize the intervention of irrelevant environmental factors [75-78,82,84,86,87,90,94,96-116,119-123,125,128]. Each data collection session involved 1 participant, who was pretrained for a few minutes before their data were collected for experimental purposes. Researchers aimed to focus participants’ attention on specific movements and perform standardized activities. The results suggest that collecting pretrained ADLs data provides standardized motion data for training ADLs recognition, but this approach might not capture the variety of behaviors necessary to accurately judge specific motion data. Meanwhile, the results also indicate that researchers concentrated on laboratory-based activities for professional use, such as the classification and identification of ADLs and the functionality of joints.

#### Study Outcome

The included studies (n=54) were all aimed at the identification and classification of activity trajectories (Figure 2). The findings indicate that most included research (n=39, 72%) focused on data analysis. Overall, 2 (4%) studies provided real-time monitoring; 3 (6%) studies offered feedback; and 10 (19%) studies were dedicated to visualization, including 6 studies that visualized original data without AI processing. The purpose of visualization is to provide professionals [88,114] or users [104,118] with the ability to view motion statuses. The visualization of motion data without classification is used in medical research [79,80,88,102,104,114], and the data are intended for use by health care professionals. Excluding the studies using animation and VR techniques, such as the one by Zelck et al [104], who proposed the animation of digital maritime workers’ working processes, and another study by Ahmed and Demirel [102], who demonstrated human performance in normal situations and aircraft accidents using VR, and the data from the latter are accessible to the public. Generally, studies focusing on ADLs primarily used data from the healthy public for professional use.

### Risk of Bias and Quality Assessment

In conducting a thorough risk of bias and quality assessment of the selected studies, our scrutiny was directed toward several critical dimensions: participant selection transparency, the accuracy and completeness of data acquisition methodologies, the analytical rigor, and the integrity in reporting outcomes. Notably, prevalent biases included selection bias, rooted in participant recruitment strategies; measurement bias, owing to sensor placement inconsistencies or activity categorization; and reporting bias. Studies using public data sets or conducted within laboratory confines typically showed reduced bias risks. In contrast, studies with constrained participant demographics or undertaken in less regulated environments faced heightened bias risks, primarily affecting data representativeness and the influence of external variables on activity assessments.

The included articles variably reported on participants’ gender and age specifics—57% (31/54) of the articles provided detailed demographic data [77-79,82,86,89-91,93,94,97-99,101,103, 105-107,109-113,115,116,119,122,124-127], while others (22/54, 41%) omitted such specifics [75,76,80,81,83-85,87,88,92,95,96,100,102,104,108, 114,117,118,120,121,123,128]. The sensor models used across studies are meticulously documented within the articles and are detailed in the Multimedia Appendix 1. It was observed that the spectrum of daily living activities discussed was somewhat narrow, seldom addressing activities that entail object interaction. The theoretical underpinning, such as that provided by ANT [68,69] and activity classification [130], lends robust support to the motion data analysis, facilitating a nuanced understanding of the data. The selected articles did not address the incorporation of verbal language or cultural customs in the context of data collection or the execution of physical activities. Thus, we consider that the concentration on physical movement to the exclusion of linguistic or cultural contexts likely accounts for the omission of cultural background in the analysis. Data collection in laboratory settings is controlled, thereby eliminating interference from factors such as socioeconomic elements. The physical tasks assigned to participants encompass routine daily movements or activities well-acquainted with athletes, effectively eliminating potential interferences from policy-related factors.

## Discussion

### Interaction Between Devices, Users, and Environmental Factors

The features of the selected studies were systematically categorized into study outcome, sensor configuration, participant types, activity types, monitoring, environment types, and AI involvement, as illustrated in NDLPA among included studies (Figure 2). This organization was informed by the visualization of Straczkiewicz et al [42], who elucidated the complex interrelations among diverse human activity recognition process phases. Drawing upon the theoretical foundation provided by ANT [64,68,69], our analysis delved into the NDLPA by investigating the dynamics of actor-led activity; purpose-oriented interaction; and interaction among devices, individuals, and environmental factors.

### Actor-Led Activity

#### Overview

Among the 54 articles selected for review, 3 main categories of initiators in health and physical wellness application research were identified: participants (including health information beneficiaries, professionals, caregivers, and interactional factors), researchers in health care, and researchers in computing. According to ANT [68,69], the interaction within the activity network is governed by those who lead the activity. As researchers are the initiators for implementing ADL research, we classified environment types by the extent of the researchers’ intervention, namely, controlled and in the wild.

#### Controlled

In controlled settings, researchers initiated the activity. Participants conducted time- and environment-restricted daily activities following the researchers’ activity assignments. Most articles mentioned that participants undergo pretraining before starting data collection to ensure that the data are typical [90,96]. Most athlete participants are in controlled environments. Researchers stated that motion data collection was controlled in specific movements, times, and spaces to guarantee optimal exercise time [81,121]. There is no other human-athlete interaction, except for 1 article using a virtual athlete [96]. This approach ensures the typicality of the data and the precision of the movement trajectories, which are essential for analyzing physical activity.

#### In the Wild

In uncontrolled or in-the-wild settings, participants are inclined to initiate activities, with interaction between participants and ambiance minimally restricted by researchers. Researchers configure sensors within the range of participants’ activities (cameras, smart water meters, pressure mats, etc), allowing participants to produce movement trajectories relating to their intents. Findings include 2 types of in-the-wild activities: one is video data sets captured in real-world situations, and the other is based chiefly on motion and multiple sensor data (eg, temperature and light use) to infer work and daily routines in living scenarios. The included studies in this review used data from in-the-wild activities, with 11 papers using data from the general population [79,80,85,88,89,91-93,95,118,126] and 1 article using data from older adults [124]. One study mentioned data collected from interactional factors, a water container [93] as the interactional initiator. Placing a motion-tracking device on the container that passively triggers humans’ drinking movements may accurately detect the trajectory of upper limb movements.

Generally, most included investigations on everyday living and physical activities have been conducted in controlled settings, where researchers aim to capture accurate and standardized movements for health professionals and activity recognition processing. Although participants receive activity guidance and practice movements before the experiment, inconsistencies in activity objectives among them may arise. Alternatively, research using activity data from in-the-wild settings could provide a more intimate and real-life interaction but generate excess data. Moreover, it can achieve data controllability when the initiators are passive objects.

### Purpose-Orientated Network

#### Overview

The activity initiator guides the purpose. As the research and the content of the activity were led by the researchers, the purpose of the research becomes the purpose of the activity in the network. We consider study outcomes, activity types, and monitoring types as elements forming the purpose of the activity networks.

#### Study Outcomes

The fields of human activity recognition and motion analysis have consistently been focal points of research, with an increasing volume of publications annually, particularly maintaining a stable high volume over the past 3 years (Figure 3). Studies have primarily focused on data visualization and monitoring, targeting daily activities and local body movements. Studies providing data feedback have addressed movement correction [123], outlier indication [113], and body posture correction [108]. It is noteworthy that outcomes related to feedback provision and data visualization have chiefly used in-the-wild data during the initial 6 years, shifting toward controlled data in the subsequent years, a transition likely influenced by advancements in research methodologies and technological evolution.

#### Activity Types

Most studies on activity trajectory recognition have focused on whole body movements, with local movements and movements related to environments being rare. Over the past 3 years, researchers have started to shift the target beneficiaries of their sports research to the general public, with physical activity education gradually overtaking professional sports. The dominant type of activities remains predominantly oriented toward daily living, with sports-related activities progressively shifted to ordinary activity.

#### Monitoring Types

Researchers described the nodes of data acquisition in terms of activity content and duration, and these nodes serve in subsequent data computation and analytical processes. They directed participants to undertake activities conforming to the predetermined criteria associated with these nodes. Moreover, research using in-the-wild data adapt their methodologies to the unpredictable nature of activity content and duration, ensuring a structured approach to capturing motion data despite the inherent variability. This nuanced guidance ensures that even in uncontrolled environments, the collection and analysis of data remain methodically aligned with the research objectives.

### Interaction Among Devices, Individuals, and Environmental Factors

Activity trajectory is demonstrated through the interaction between humans, sensors, and environments. Motion-tracking devices, sensor positions, and environments factors are the main features to be evaluated.

#### Motion-Tracking Devices

Given this study’s basis on motion tracking technology and the categorization of sensor types, we divided device types into complex devices (multiple sensors, eg, temperature, lighting, pressure accelerometer, and gyroscope sensors, VR, AR, etc) [91,92,101,113]; portable devices (smartphone or smartwatch) [77,79,81,92]; basic devices (motion-tracking sensors, eg, accelerometer and gyroscope sensors) [78,79,84,90,93]; and environmental devices (other sensors, eg, cameras, radiofrequency ID tags, etc) [100,102,117,124,128]. The configuration of complex devices aims at research related to local motions, complex motions, and environmental interactions. In conditions using complex devices, participants wore portable devices such as smartwatches to record motion data simultaneously with built-in multiple sensors (eg, ambient light sensor, UV light exposure sensor, and skin temperature sensor) [81]. A portable device with built-in accelerometer and gyroscope sensors performed a similar motion tracking task as a basic device. Most of them were used to determine everyday activities’ trajectories as they are easy to perform. In conditions using portable and environmental devices, the portable device was placed on the body, and the participant activated sensors placed in the environment. The active and passive motion data form an activity network [92]. Accelerometer and gyroscope sensors were placed on multiple areas of participants’ bodies to monitor their full-body motions. However, using cameras to capture athletes’ movements and environmental devices (eg, pressure mats, mercury contacts, and float sensors) [91] to record living routine is favored by researchers.

#### Positions of Sensors

Our study categorizes device placements by areas of the human body and their relative distance from the human body, namely, in physical spaces, on objects, on the full body, on the upper limb, and on the lower limb. The results indicate that sensor placement is related to the study’s purpose and the intensity of the activity. Sensors used in physical spaces or on the full body examined high-intensity activities (eg, professional sports), correlating with accurate data acquisition. High-intensity activities, such as running [105] and throwing darts [110], were primarily studied using devices on local body areas. Devices placed on specific objects received limited daily exercise data but were consistent with the study’s objectives. Sensors attached to different areas of the participants’ bodies collected data from those areas and integrated with participants’ movements. Sensors placed in the environment passively received signals, yet timing and activity patterns could be detected.

#### Environmental Factors

The environments for study implementation were mainly controlled by researchers. The movement trajectory of participants’ performance aligned with the research objectives. The content and duration of activities were fixed, and devices were placed according to the research purposes. Participants passively interacted with their surroundings. Performing daily living or physical activities in an experimental setting might influence the participants’ intent of the action. In these settings, participants’ movements were unrelated to habitual actions. The environment and devices reflected interactions with researchers but did not interact with the participants, leading to a severed connection between the activity network and the participant involved. However, in video recording scenarios, participants performed activities in real-life settings, potentially providing purposeful and personalized movement data for research.

### Investigation Process

Activity identification and recognition have been the primary focus of research on ADLs and physical activities in health and wellness applications over the past decade. The fundamental objectives are to enhance the accuracy of motion tracking data recognition and its practical use for health professionals. From the perspective of NDLPA, researchers acted as initiators of activity networks, leading the content and environments of activities in most activity recognition studies. Participants provided standard movement data within a restricted time and environment, lacking personal purpose and awareness of real-life activity. Participants did not fully become the initiators of the activity networks, but real-life activity data could be more complex. The variety of activity scenarios, user-friendly sensor design, data collection at specific time points, and data variances produced by complex activities and various real-life purposes could present research opportunities for studying daily living and physical activities for health and physical wellness applications. Meanwhile, designers have the opportunity to engage in designing user-initiated activities and collect technology-based motion data for health and physical wellness application research.

**Figure 4:**
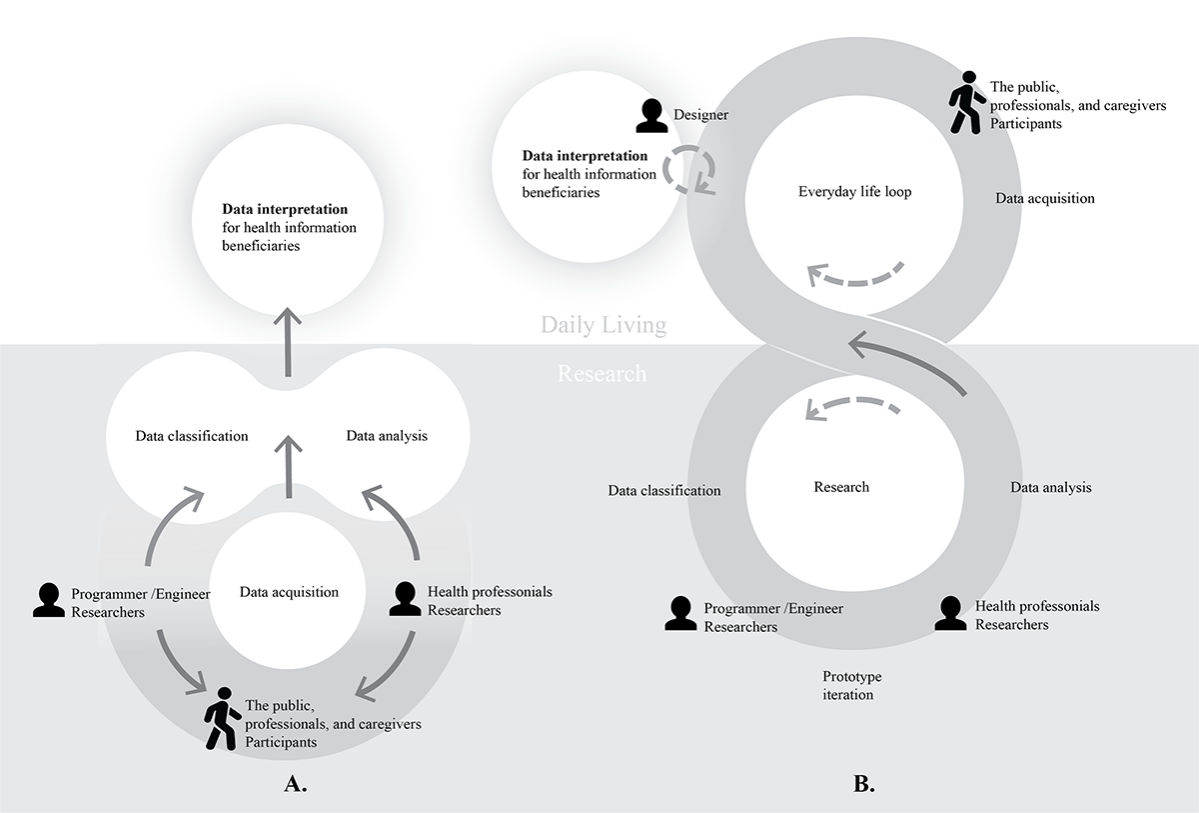
(A) The investigation process of motion data on daily living and physical activity for health care application and (B) a designer-involved investigation process of motion data on daily living and physical activity for health care application based on data-enabled design.

According to the descriptions in the included articles, the investigation of ADLs and physical activities in health care applications covers 4 phases: data acquisition, data classification, data analysis, and data interpretation, as shown in Figure 4A. In the process of motion tracking data acquisition, researchers recruited participants and conducted data collection. Participants performed assigned activities under full or partial surveillance by researchers. The data were supervised and categorized into standardized movements corresponding to specific activities. At this stage, the activities varied by different engaged areas on participants’ bodies but not by the content of daily living activities. Researchers trained various algorithm models for data classification to achieve high activity recognition accuracy and used different data sets to test the system’s validity. In the data analysis stage, researchers identified standardized activities and examined the difference values through motion tracking parameters. Using these parameters, researchers analyzed activity norms and behaviors. This stage is also associated with data monitoring. Data classification and analysis can be combined or conducted independently for different research evaluation purposes. Data interpretation, such as data visualization and feedback provision, was created by researchers from the health, engineering, and computing domains. It interacted with health information beneficiaries (eg, professionals, users, and caregivers). The health information was presented in either video or image forms for visualization or in text forms for feedback to users.

On the basis of the investigation process of ADLs and physical activities in health care applications, participants’ involvement occurs at an early stage. However, participants are situated in a controlled environment for experimental purposes, with devices attached to their bodies without active interaction. Although the data are clear for classification and analysis, they rarely explain the diversity of ADLs. In the data interpretation stage, there is a weak connection between the data and the general public owing to the dominant purpose of activity identification research aimed at professional use. Designers can aid in bridging communication between information and the public. As described in the literature in previous sections, human motion data are essential components of activity recognition and analysis [1,2], with the public being the beneficiary of physical activity intervention research [11,12]. We propose that designers, coupled with researchers’ guidelines, participate in the investigation and application process, which promises to facilitate data collection and interpretation.

### Designer-Involved Investigation Process

The research of health care and physical wellness applications is directed toward professional deployment and public health enhancement. However, it has been observed that there exists a gap between the motion data collected from participants and their applicability to real-life situations. In addition, research in health care applications often fails to encompass the breadth of data diversity; there is a noticeable lack of meaningful engagement between devices and participants, and the process of making data understandable for the public is fraught with difficulties. To address these issues, we advocate for a research methodology that integrates DED framework [61-63], a designer-involved research process of motion data on ADLs and physical activity for health care application research, as illustrated in Figure 4B.

In this proposed methodology, designers collaborate with health care researchers to understand the specific information required for participant recruitment in a given project. They assist health care researchers in designing data collection strategies while considering the daily behavior patterns of the target population, thereby refining the data collection plan. Researchers from computing and engineering fields undertake the collection and categorization of data. These data span various demographics, purposes, devices, and scenarios from designers and are tailored for a multitude of applications. Such research process encompasses a cycle of prototype iterations. Researchers from the health field analyze classified data, identify abnormal motion data, and then provide guidelines for designers. Designers, positioned as intermediaries between users and researchers, leverage these guidelines and their nuanced understanding of human daily experiences including participant involvement and activity motivation [131] to reinterpret professional data into user-friendly formats. Furthermore, designers play a pivotal role in collecting motion data that reflect a diverse array of demographics, intentions, devices, and scenarios. They are able to simplify the data and assist with data interpretation for the recipients of health information.

The integration of design principles into the development of health care applications is key to enhancing user engagement, offering more intuitive interfaces that potentially increase adherence to health interventions through user-friendly designs. Our findings indicate that in intricate settings, standardizing data to mitigate external variables might lead to binary misjudgments. Given that daily activities are influenced by individual attributes, environments, and habits, the role of designers becomes critical in tailoring solutions to meet the varied needs of distinct user groups. They fine-tune the implementation methods and locations for motion-tracking devices, such as using cameras or accelerometer and gyroscope sensors and determining specific areas for deployment, such as full-body tracking or environmental interaction. They also specify the nature of the data to be collected, differentiating between event-driven data, which capture specific actions such as walking across varied material surfaces, and time-sequential data, which records information over continuous periods such as walking at night. This process enhances the accuracy and applicability of collected information, facilitating the development of data-driven products that reflect real user experiences. During the experiment, the dialogue between users and designers’ aids in identifying actual and hidden needs, thus refining information precision and application. Upon analyzing the data, health care researchers collaborate with designers to reformat health information into accessible, engaging formats on digital interfaces, using techniques such as digital twins, animations, visualizations, and gamification. This method enhances user engagement, elicits valuable feedback, and fosters ongoing improvement in data accuracy, thus promoting a cycle of continuous information enhancement.

In addition, designers can assist engineers in designing user-friendly devices and facilitate actuator developments to provide users with timely feedback. The iterative refinement of technology that monitors ADLs and physical activity motion data in health application research is driven by the diversity of the population, necessitating continuous optimization. Design’s contribution is pivotal in promoting effective communication, nurturing relationships, and pursuing sustainable innovation. By involving designers in the process, the development of activities initiated by users and the creation of user-friendly motion-tracking sensors are significantly improved. This, in turn, supports the cyclical development of health and physical wellness applications [64,65], ensuring that they are more aligned with real-world needs and contexts. Thus, in the sphere of health care and physical wellness application research, the involvement of designers is crucial for the effective acquisition and interpretation of data, ensuring that the data are relevant and beneficial in everyday scenarios.

Designers’ involvement is essential in shaping design and implementation strategies, ensuring that technology solutions are tailored to meet the inherent human needs for interpreting health-related information. This approach is instrumental in improving the monitoring and management of health conditions, thereby improving patient outcomes. This not only improves usability but also ensures that technologies are perceived as useful and enjoyable, mirroring the foundational criteria of the TAM [54,55]. However, potential challenges include ensuring effective communication and collaboration across multidisciplinary teams, aligning different objectives and methodologies, and the complexity of translating complex health data into accessible and actionable information for users.

### Limitations and Future Directions

This review process identified key limitations and future research directions in the use of motion tracking technologies for health care. It pinpointed risks of selection and measurement biases due to participant recruitment strategies and inconsistencies in sensor placement or activity categorization. Highlighting the inadequate exploration of feedback mechanisms, this review emphasized the necessity for future studies to optimize user communication. The use of sensors for motion data collection offers a method to preserve privacy, contrasting with cameras or recorded videos, which might inadvertently compromise it. One included study introduced a Kinect and smartwatch system, focusing on health care professionals’ privacy and showcasing a strong commitment to privacy and ethical considerations in system design [79]. The minimal focus on privacy protection in other included studies indicates a potential research gap. Furthermore, this review underscored the unexplored potential of integrating geolocation data into physical activity interventions, proposing future research to delve into scenario-based health care applications. The investigation did not fully address the influences of cultural, socioeconomic, and policy-related factors on physical activity, or the budget constraints that may have curtailed the exploration of these dimensions. Moreover, it acknowledged the potential publication bias and the influence of study quality on the findings, especially given researchers’ inclination to report positive outcomes of health care application development. Although the search included major databases, there is a possibility that relevant studies in other databases were overlooked. In addition, database search confined to English language might have excluded pertinent non-English studies. These areas present fertile ground for future research to ensure a more holistic understanding and application of motion tracking technologies in diverse settings.

### Implications

This study significantly contributes to future research by demonstrating the importance of a multidisciplinary approach in health care application development, particularly in the integration of design principles with engineering and health sciences. It lays a foundation for further exploration into how designers can enhance the usability and effectiveness of health applications through user-centric design and data interpretation. Future studies can build on this framework to investigate specific design strategies and their impact on user engagement, adherence, and overall health outcomes, potentially leading to more personalized and effective health interventions. For real-world applications, this approach could fundamentally transform the development of health care apps, making them more accessible, intuitive, and tailored to individual needs, ultimately leading to improved health outcomes and patient engagement.

### Conclusions

The study systematically reviewed the implementation of motion tracking in health care application research, revealing a discrepancy between the data collection and its effective application in real-world scenarios. Informed by ANT, we explore the dynamics of actor-led activities and purpose-oriented interactions, focusing on participants, sensors, cameras, and environmental factors in health care research concerning ADLs. The guiding roles of activity initiators significantly influence the phases of data acquisition, classification, analysis, and interpretation, underscoring the potential to enhance the accuracy of motion tracking and activity recognition when considering contextual factors. By advocating a designer-involved research process of motion data on ADLs and physical activity for health care application research, this study emphasizes the essential role of incorporating design principles via a DED framework in developing health care applications. This integration is crucial for aligning motion data collection with practical, real-life applications. By adopting a multidisciplinary approach and combining insights from design, engineering, and health sciences, the research demonstrates potential pathways for making health applications more user-friendly and effective. It underscores the necessity of engaging designers in the research process to ensure that health technologies are accessible, intuitive, and tailored to meet the diverse needs of users. Designers play a key role in customizing the deployment and data collection methods of motion-tracking devices, which improves the relevance and accuracy of collected data, leading to better-informed health applications. The iterative dialogue between users and designers during development refines the precision of information and its application, fostering a cycle of continuous improvement. By transforming complex health data into engaging, understandable formats, designers help translate user needs into actionable health solutions, promoting better monitoring and management of health conditions. The study advocates for further research to explore and refine these integrations, offering a direction that promises to transform health care monitoring and interventions, ultimately enhancing patient outcomes and engagement.

## Appendix

Multimedia Appendix 1 Summary of included studies based on participants, interventions, comparisons, and outcomes methodology.

Multimedia Appendix 2 Search terms and strategies based participants, interventions, comparisons, and outcomes methodology and research questions (including 3 phases).

Multimedia Appendix 3 The Network of Daily Living and Physical Activity (NDLPA) among included studies. AI: artificial intelligence.

Multimedia Appendix 4 PRISMA checklist.

## Abbreviations

ADL: activity of daily living
AI: artificial intelligence
ANT: actor network theory
DED: data-enabled design
IoT: Internet of Things
NDLPA: Network of Daily Living and Physical Activity
PICO: participants, interventions, comparisons, and outcomes
TAM: technology acceptance model
VR: virtual reality
