# Supplementary figures and images for: Motion Tracking of Daily Living and Physical Activities in Health Care: Systematic Review From Designers’ Perspective
Source: JMIR Mhealth Uhealth. 2024 May 6;12:e46282. doi: 10.2196/46282 (PMC11106703; doi:10.2196/46282)

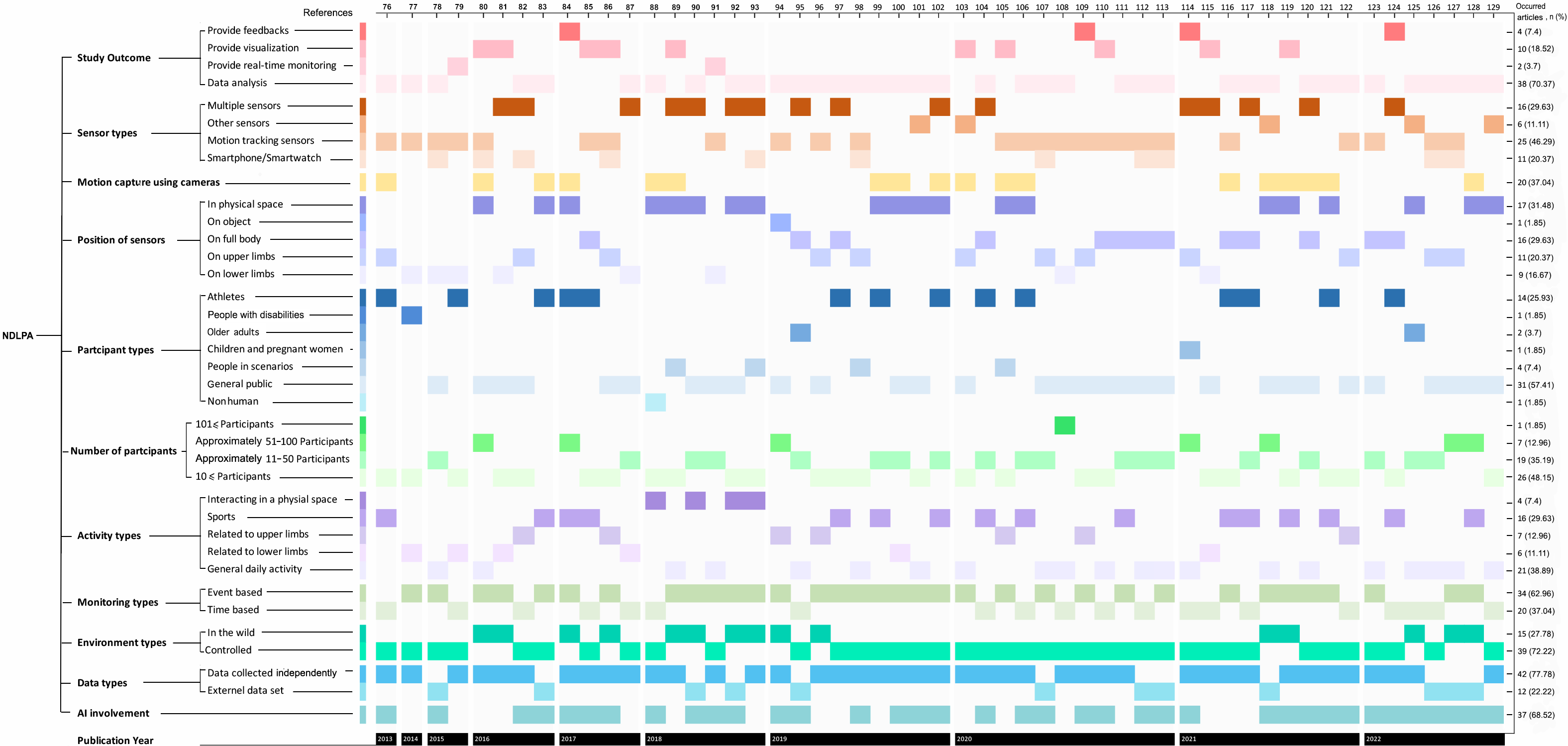

Supplement: Multimedia Appendix 3 [file mhealth_v12i1e46282_app3.png]
